# A Randomised, Cross-Over, Placebo-Controlled Study of *Aloe vera* in Patients with Irritable Bowel Syndrome: Effects on Patient Quality of Life

**DOI:** 10.5402/2011/206103

**Published:** 2010-10-11

**Authors:** H. A. Hutchings, K. Wareham, J. N. Baxter, P. Atherton, J. G. C. Kingham, P. Duane, L. Thomas, M. Thomas, C. L. Ch'ng, J. G. Williams

**Affiliations:** ^1^School of Medicine, University of Wales Swansea, Singleton Park, Swansea SA2 8PP, UK; ^2^Clinical Research Unit, Morriston Hospital, Swansea NHS Trust, Swansea SA66NL, UK; ^3^Morriston Hospital, ABM NHS Trust, Swansea SA66NL, UK; ^4^Forever Living Products (UK) Ltd, Warwick, W346RB, UK; ^5^Singleton Hospital, ABM NHS Trust, Swansea SA28QA, UK; ^6^Neath Port Talbot Hospital, ABM NHS Trust, Port Talbot SA127BX, UK

## Abstract

*Background*. Irritable bowel syndrome (IBS) is a chronic, difficult to treat condition. The efficacy of *Aloe vera* in treating IBS symptoms is not yet proven. The purpose of this study was to determine if *Aloe vera* is effective in improving quality of life. *Methods*. A multicentre, randomised, double-blind, cross-over placebo controlled study design. Patients were randomised to *Aloe vera*, wash-out, placebo or placebo, washout, *Aloe vera*. Each preparation (60 mL) was taken orally twice a day. Patient quality of life was measured using the Gastrointestinal Symptoms Rating Score, Irritable Bowel Syndrome Quality of Life, EuroQol and the Short-Form-12 at baseline and treatment periods 1 and 2. *Results*. A total of 110 patients were randomised, but only 47 completed all questionnaires and both study arms. Statistical analysis showed no difference between the placebo and *Aloe vera* treatment in quality of life. Discussion. This study was unable to show that *Aloe vera* was superior to placebo in improving quality of life. Drop outs and other confounding factors may have impacted on the power of the study to detect a clinically important difference. *Conclusion*. This study failed to find *Aloe vera* superior to placebo in improving quality of life proven Irritable Bowel Syndrome patients.

## 1. Introduction

Irritable bowel syndrome (IBS) is a functional bowel disorder in which abdominal discomfort or pain is associated with a change in bowel habit, and with features of disordered defecation [1]. Some sufferers may have coexisting anxiety or depression [2, 3].

Community-based studies have highlighted that persons with IBS have greater disability and a threefold higher absenteeism from work than do healthy controls leading to greater health care utilisation [4–7]. It has been suggested that psychosocial distress in IBS patients influences health-care seeking behaviour [8–10]. Patients with IBS also have poorer quality of life than persons without the disorder [4–6]. 

Surveys of Western populations have revealed prevalences of IBS between 15–20% with a higher prevalence in women [8]. However, despite its prevalence, there remains a proven lack of safe efficacious medical treatments for IBS [11]. Conventional treatments include antispasmodics, antidiarrheals, antidepressants, and anxiolytics medications [1, 2, 12, 13]. A review of randomized, controlled trials found no convincing evidence to support the efficacy of any medication but highlighted flaws in the methodology of the trials [14]. A more recent review found that some treatments were beneficial for individual symptoms but highlighted the fact that treatment should be targeted at the major symptoms [13]. No single treatment appears to be effective in relieving all the symptoms associated with the disorder. 

Growing numbers of patients have turned to complementary and alternative medicine (CAM) after failing to find adequate relief from conventional remedies [15, 16]. It has previously been reported that usage of alternative treatments was greater for IBS than some other gastrointestinal disorders and that up to 41% of IBS patients would consult an alternative practitioner if conventional remedies failed [17, 18]. Alternative treatments for IBS include hypnosis [11, 19], acupuncture [15], cognitive behaviour therapy [20], yoga [2], and herbal remedies (such a peppermint oil [21] and Chinese herbal medicines [22]) which have been associated with varying degrees of success.

The *Aloe vera* plant is a perennial succulent with thick leaves whose central pulp consists of large, thin-walled cells containing a thin clear jelly-like *Aloe vera *gel [23]. There has been a growing commercial interest in *Aloe vera* gel, which is used both topically in cosmetic products and herbal remedies and drinks [24]. Externally *Aloe* products have been used for treatment of wounds, burns, and skin irritations. *Aloe Vera* drinks have been promoted for constipation, coughs, wounds, ulcers, diabetes, cancer, headaches, and many other [23, 25]. Although there is evidence of efficacy of *Aloe vera* in some conditions, there is limited data available for most conditions [25]. * Aloe* is commonly used in IBS, particularly the constipation-predominant subtype [26]. 

Although it is considered “safe” there are currently limited data regarding the efficacy of *Aloe vera* in the treatment of IBS. A study assessing a compound containing celandine, *Aloe vera* and psyllium in the treatment of constipation resulted in an greater symptom improvement in the active group when compared to placebo [27]. There have been no large randomised controlled studies assessing the action of *Aloe vera* alone in a cross-over fashion. The purpose of this study was therefore to assess the efficacy of an *Aloe vera* gel drink compared with a placebo in patients with recognised IBS.

## 2. Methods

### 2.1. Study Design

The study was a multi-centre prospective randomised, placebo-controlled, double-blind, cross-over study. The study took place in three hospitals in South West Wales, UK. Following screening, patients were randomly allocated into one of two groups. Following baseline assessments patients entered a two-week screening period followed by a five-month treatment period (part 1). At the end of this period there was a two week wash-out period. Patients then entered a second five-month treatment period when they received the alternative treatment (part 2). Group AB consisted of an *Aloe vera* drink 60 mls twice daily for five months followed by two weeks wash out then matched placebo drink for 60 mls twice daily for five months. Group BA consisted of placebo for five months, two week washout, then *Aloe vera* for five months. Both *Aloe vera* and placebo drinks were identical in taste and produced by the same manufacturer. The study was approved by the Local Research Ethics Committee.

### 2.2. Patient Selection

Inclusion criteria were male or female patients aged at least 18 years, diagnosed as suffering with Irritable Bowel Syndrome (IBS) according to the Rome II criteria [1] for at least one year and had received previous treatment; were complaining of abdominal pain; were seeking further treatment; were able and willing to give consent to be randomised into the study. Female patients were included if they were taking adequate contraceptive precautions and had a negative pregnancy test at baseline. Patients on medication for stable medical conditions not considered to exert an effect on the study intervention were eligible for recruitment. Certain medications for relief of diarrhoea or constipation were permitted. Patients were excluded if they had significant other GI tract disease, had undergone previous GI tract surgery, were known to be defaulters at clinic and might be difficult to follow up, were on drugs which might affect motility, or had a current or recent history of drug or alcohol abuse.

### 2.3. Study Evaluation and Statistical Tests

The primary outcome measure was patient quality of life. Four quality of life scales were used in the study completed by patients at baseline, end of study period 1 (5 months) and end of study period 2 (10 months). Compliance with treatment was assessed by measuring the number of returned empty containers at the end of each study period.

#### 2.3.1. Gastrointestinal Symptoms Rating Scale (GSRS) [28, 29]

This is a disease-specific quality of life questionnaire. The scoring system originally devised for measuring changes in psychopathology, has been modified for use in patients with IBS and peptic ulcer disease. The GSRS scoring system has been evaluated in the UK and Scandinavia. Four subscales (abdominal pain syndrome, dyspeptic syndrome, indigestion syndrome, bowel dysfunction syndrome) and a total gastrointestinal symptoms score were calculated from the GSRS.

#### 2.3.2. EuroQol (EQ5D) Questionnaire [30, 31]

EQ-5D is a generic instrument for use as a measure of health outcome. Applicable to a wide range of health conditions and treatments, it provides a simple descriptive profile and a single index value for health status. EuroQol assessment consisted of a descriptive score based on 5 health dimensions together with a self-rated visual analogue score.

#### 2.3.3. Short Form 12 (SF 12) Quality of Life Questionnaire [32]

This is a shortened version of the generic SF36 questionnaire [33]. It has been well validated and is considered to be almost as efficient as the full version for assessing patients' quality of life [32, 34]. Two summary scores were calculated from the SF-12, a Physical Component Summary Scale Score (PCS), and a Mental Component Summary Scale Score (MCS).

#### 2.3.4. The Irritable Bowel Syndrome Quality of Life Questionnaire (IBSQOL) [35, 36]

This is a disease-specific quality of life assessment for patients with Irritable Bowel Syndrome. The IBSQOL questionnaire produced 8 subscales scores for each patient: emotional health, mental health, sleep, energy, physical functioning, diet, social role, and physical role.

### 2.4. Statistical Analysis

Independent sample *t*-tests or Chi-square tests were used prior to any treatment at baseline to check that the two treatment groups were comparable. Data for patients who were randomised into a group and then failed to continue after visit two were assessed on an intention to treat basis, that is, within their original treatment allocation group. Statistical analysis was carried out to differentiate any genuine treatment effects from possible “carry-over” effects introduced by the different order in which the patients received the study treatment. Data were analysed according to statistical procedures for full cross-over trials as recommended by Armitage and Berry [37] when there was no strong carry-over effect. Cross-over comparisons were based on those subjects with data for the end of treatment period one and the end of treatment period two (i.e., at 5 months and 10 months).

#### 2.4.1. Power of the Study

When analysed as a full crossover study, a sample size of 55 per group would have an 80% power to detect a difference of 0.37 SD (12 points in patient quality of life scores) at a significance of 0.05. If necessary, when analysed as a simple randomised trial, a sample size of 55 per group would have an 80% power to detect a difference of 0.54 SD (16 points in patient quality of life scores) at a significance of 0.05.

## 3. Results

**Figure 1:**
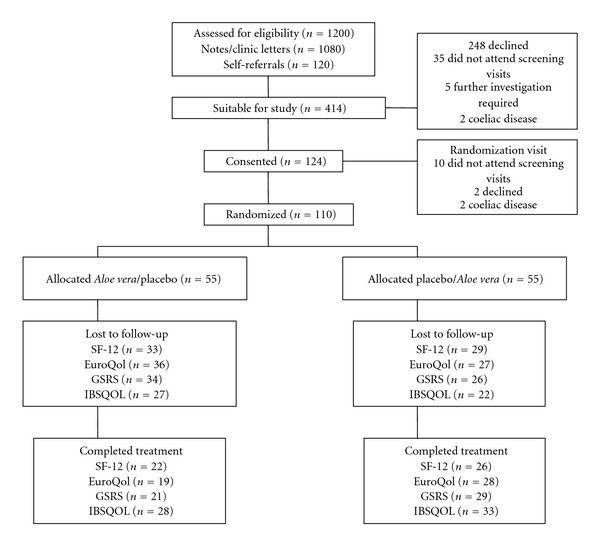
Patient progress through the stages of the trial.

During the period from 1st August 2001 to 31st July 2003 a total of 1080 patients notes/clinic letters from three hospitals in South West Wales, UK (Morriston Hospital, Singleton Hospital and Neath Hospital) were screened to identify suitable patients. A further 120 patients were screened following self-referral via responses to posters in GP practices. Letters were sent to 414 of these patients inviting them to discuss the study. A total of 124 patients consented to enter the study of which 110 were randomised. Figure 1 shows a flow diagram of the patient recruitment in the study.

Baseline analysis of the data highlighted that the two groups were well matched and that there were no significant differences between the two groups with respect to their demography or symptoms (Table 1).

### 3.1. Comparison of Quality of Life Scores

When taking into account the ordering of the treatments, there were found to be no significant differences between any of the scores for the generic or disease-specific scales based on the ordering that the treatments were received (*P* > .05 for all comparisons). Similarly there were found to be no treatment period effects for any of the scores on the generic or disease-specific scales (*P* > .05 for all comparisons).

**Figure 2:**
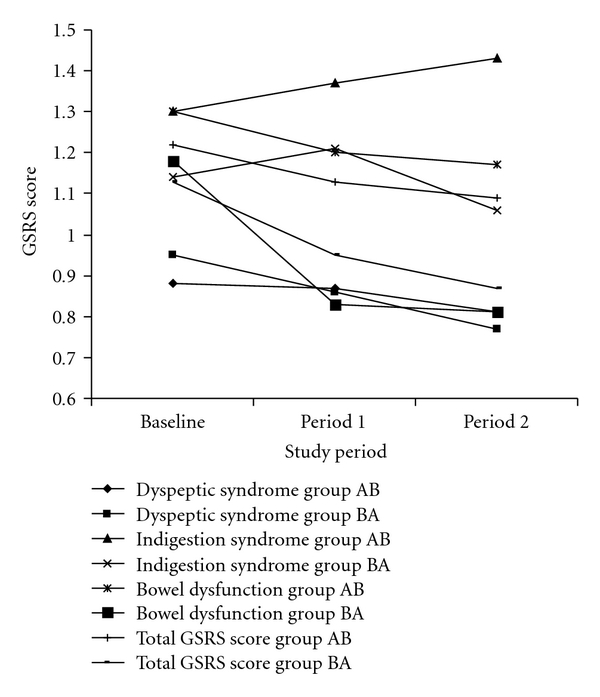
Gastrointestinal Symptoms Rating Scale Questionnaire (GSRS) Dyspeptic syndrome, Indigestion syndrome, Bowel dysfunction and Total scores for each study group at baseline, period 1 (5 months) and period 2 (10 months).

**Figure 3:**
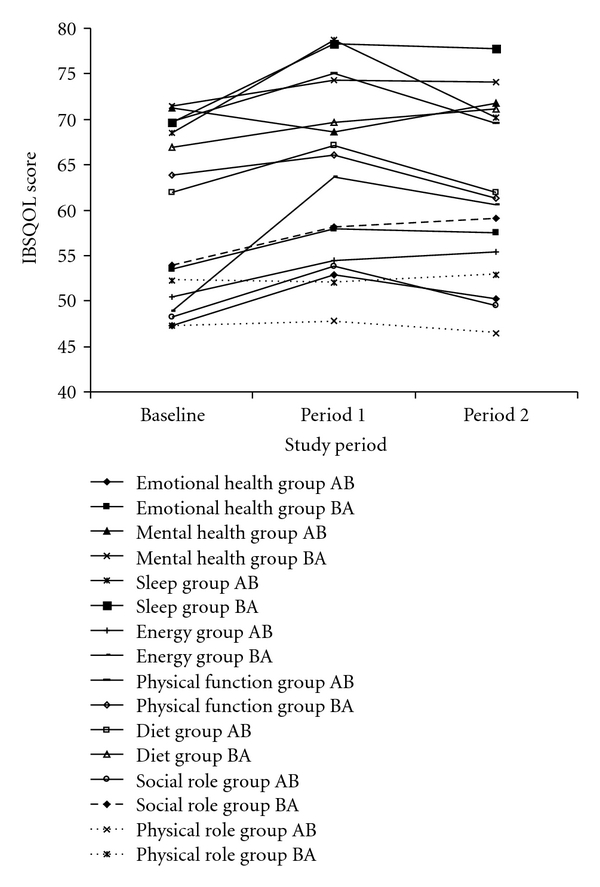
The Irritable Bowel Syndrome Quality of Life questionnaire (IBSQOL) Emotional health, Mental health, Sleep, Physical function, Diet, Social role and Physical role scores for each study group at baseline, period 1 (5 months) and period 2 (10 months).

As there were no significant ordering or treatment period effects, the results for each scale were analysed as a full cross-over study. The cross-over analysis identified that there were no significant differences between treatment A and B with respect to the generic SF-12 or EQ-5D scores (*P* > .05 for all comparisons). Similarly, no significant differences were found between treatment A and B with respect to the disease specific scores generated from the GSRS and IBSQOL (*P* > .05 for all comparisons). Figures 2 and 3 illustrate the mean scores for the GSRS (dyspeptic syndrome, indigestion syndrome, bowel dysfunction, and total GSRS score) and IBSQOL (emotional health, mental health, sleep, energy, physical function, diet, social role and physical role) for each treatment period.

### 3.2. Compliance

Both study groups were fully compliant with the assigned treatments with all treatment containers being returned empty.

## 4. Discussion

This study failed to find *Aloe vera* superior to placebo in improving any aspect of patient quality of life using either disease specific or generic quality of life measures in this group of patients with proven Irritable Bowel Syndrome (IBS) (according to the Rome II Criteria [1]). Compliance with treatment in those who entered and or completed both arms of the study appeared to be acceptable as determined by the number of returned empty containers.

Poor recruitment numbers, a large number of patients withdrawing from the study and the long treatment period all may have had an effect on the results. In addition, although over 1000 patients were initially identified with symptoms suggestive of IBS only about 10% of these were recruited, which could mean that our sample was not truly representative of the broad spectrum of IBS patients. All patients who entered the study did however meet the Rome II criteria [1] for IBS so were considered appropriate. 

The need for patients to discontinue certain medications prior to entry into the study was unacceptable to some patients and again impacted on recruitment. The reduced recruitment rate affected the power of the study and was further compounded by the number of patients who discontinued throughout the study for a variety of reasons which included an increase in symptoms in both groups but more marked in the *Aloe vera* arm of the study. The final sample was only sufficient to detect relatively large differences in quality of life scores between the two groups. A larger sample with fewer drop outs may have detected some difference between the two groups. Our results mirror those reported in a small study of patients with ulcerative colitis which was unable to detect any differences in quality of life scores following *Aloe vera* treatment [38].

Although a great deal of time was spent recruiting and discussing the study with patients this did not prevent a large number of nonattendances at randomisation and throughout the study. Attempts by mail and telephone did not elicit a response from some patients. The long duration of the study and the completion of a comprehensive daily diary card throughout the study may have resulted in the loss of some patients. The visits were fairly well spaced out and there was some flexibility in attendance days and time of day so we do not consider actual attendance would have had a great impact on preventing patients taking part in the study.

IBS is a functional gastrointestinal condition that has many components, which make it difficult to assess and treat [11, 39]. IBS can be associated with many symptoms including diarrhoea, constipation, abdominal pain, nausea, bloating, unsatisfactory defaecation, and exhaustion, with patients suffering some or all at different times. This mixed symptomatology makes treatment difficult with orthodox medicine [40]. Various approaches have been adopted for the treatment of IBS ranging from drugs to dietary modifications and counselling. The complex nature of IBS may mean that certain subpopulations of patients with IBS could benefit from *Aloe vera*. In some sufferers there is considered to be a substantial emotional and psychological component to IBS again making compliance difficult. It may be that patients whose symptoms are more physical have a better response in respect of quality of life. However, due to our reduced sample size, our study was not sufficiently robust to detect this or to fully determine which subpopulations of IBS might benefit. The disorder is also characterised by spontaneous relapse and remission and there is a recognised strong placebo response to treatment [14]. Both the *Aloe vera* and placebo arms showed improvements in some quality of life scores during the treatment periods although the differences were not sustained or significant. The placebo response to symptoms of irritable bowel syndrome has been previously documented and the duration and degree of this effect may have had an impact on our ability to separate the two treatments [39, 41, 42]. It may also be that the individuals recruited to the study are strongly in favour of alternative medicines with a strong belief in the therapies and a commitment to the concept that they will work [43, 44]. In these individuals the placebo response is likely to be even more exaggerated.

The study used a cross-over design with patients being their own control in an attempt to try and reduce some of these effects. This design in itself was associated with problems, predominantly large numbers of drop outs. Although we feel that a cross-over study was the most appropriate design, the requirement for a long study duration and the associated problems of patient retention especially for a disorder as complex as IBS limits its potential. A more conventional parallel group design may have been more useful in terms of retaining patients. The statistical analysis was undertaken on patients who completed both sequences and this resulted in a further loss of subjects. An assessment of generic and disease-specific quality of life questionnaires showed no significant difference between the *Aloe vera* and placebo group with respect to improvement of symptoms. Caution must be used when interpreting these findings as the total patient numbers are small and the fluctuating nature of the disorder could also affect the results. The treatment periods themselves were of fairly short duration for a disorder of this nature and it could be that a longer treatment period would counteract some of these effects. Other authors have documented that management of IBS requires long term involvement of the patient as no single treatment has been shown to be predictably effective [45].

At the time of the study we were not aware that some patients might experience increased symptoms on commencing *Aloe vera*, and in practice the dose is titrated down and then up again which from discussion with the manufacturer of the *Aloe vera* often resolves the issue. Unfortunately as the duration of each arm of the study was only five months this was not considered appropriate but in hindsight might have prevented some of the withdrawals.

It has been noted that the severity of patient symptoms and their effects on patient quality of life should guide treatment and that a comprehensive and multifaceted approach to treatment is required [12].

This study has therefore failed to demonstrate any benefit for *Aloe vera* in IBS, but it has also highlighted the difficulty of randomised trials in this condition. We would recommend further studies addressing some of the shortfalls of the current study including a more flexible treatment approach, longer treatment duration, and larger sample size.

## 5. Conclusions

This study failed to find *Aloe vera* superior to placebo in improving patient assessed quality of life (using two generic and two disease-specific quality of life tools) in a group of patients with proven Irritable Bowel Syndrome (according to the Rome II Criteria [1]).

## Figures and Tables

**Table 1:** Baseline characteristics of the study population by randomisation group.

Description	Group		
	*Aloe vera*/Placebo	Placebo/*Aloe vera *	*P*-value
Number	55	55	
Sex (F : M)	42 : 13	42 : 13	>.05
Mean age (SD) yr	46.0 (13.6)	47.0 (13.7)	>.05
Mean weight (SD) kg	73.1 (17.4)	74.5 (16.6)	>.05
Mean height (SD) cm	164.4 (9.4)	165.4 (10.3)	>.05
Smokers, *n* (%)	10 (18)	14 (26)	>.05
Alcohol Y : N	36 : 19	34 : 21	>.05
Employed, *n* (%)	32 (58)	26 (47)	>.05
Main IBS symptom, *n* (%)			
Pain	19 (35)	25 (46)	>.05
Bloating	6 (11)	10 (18)	>.05
Constipation	7 (13)	3 (6)	>.05
Diarrhoea	14 (26)	12 (22)	>.05
Alternating diarrhoea/	9 (16)	5 (9)	>.05
constipation			
Approx length of symptoms			>.05
per episode, *n* (%)			
0–3 days	18 (33)	16 (29)	
4–7 days	9 (16)	8 (15)	
>7 days	28 (51)	31 (56)	
Approx length of time			>.05
between episodes, *n* (%)			
<2 weeks	48 (87)	42 (76)	
2–4 weeks	5 (9)	9 (16)	
>1 month	2 (4)	4 (8)	
Fibre intake, *n* (%)			>.05
Low	13 (24)	12 (22)	
Medium	24 (44)	26 (47)	
High	17 (32)	17 (31)	
Mean systolic blood	132.2 (18.8)	132.5 (17.8)	>.05
pressure (range) mmHg			
Mean diastolic blood	83.2 (13.1)	81.5 (11.0)	>.05
pressure (range) mmHg			
Mean heart rate (range)	71.9 (10.8)	74.1 (10.0)	>.05
bpm			
